# Identification of endoplasmic reticulum-shaping proteins in *Plasmodium* parasites

**DOI:** 10.1007/s13238-016-0290-5

**Published:** 2016-08-02

**Authors:** Sha Sun, Li Lv, Zhi Yao, Purnima Bhanot, Junjie Hu, Qian Wang

**Affiliations:** 1 Department of Genetics and Cell Biology, College of Life Sciences, Nankai University, and Tianjin Key Laboratory of Protein Sciences, Tianjin, 300071 China; 2 Department of Immunology, School of Basic Medical Sciences, Tianjin Medical University, and Tianjin Key Laboratory of Cellular and Molecular Immunology, Tianjin, 300070 China; 3 Department of Microbiology and Molecular Genetics, Rutgers New Jersey Medical School, Rutgers, The State University of New Jersey, Newark, NJ 07103 USA; 4 National Laboratory of Biomacromolecules, Institute of Biophysics, Chinese Academy of Sciences, Beijing, 100101 China

**Dear Editor,**

In eukaryotic cells, the endoplasmic reticulum (ER) is a continuous membrane system involved in many critical cellular processes, including protein synthesis, lipid synthesis, and calcium storage. Morphologically, the ER is composed of cistern-like sheet structures and a reticular network of tubules. Classes of integral membrane proteins that shape the ER have been identified: the reticulons and DP1/Yop1p proteins generate ER tubules by inducing high curvature in the membrane (Voeltz et al., 2006), the atlastin GTPases and Sey1p/RHD3 proteins mediate fusion of ER membranes, forming a tubular network (Hu et al., 2009; Orso et al., 2009), and Climp63, Kinectin, and p180 play a role in stabilizing ER sheets (Shibata et al., 2010). Mutations in the determinants of ER tubules cause growth defects, short root hairs, and a neurodegenerative disease called hereditary spastic paraplegia (HSP) (Hu et al., 2009), and sheet-formation proteins are strongly upregulated in professional secretory cells when ER sheet expansion is needed, suggesting that ER morphology is tightly associated with its physiological functions (Shibata et al., 2010).

ER morphology has rarely been studied in protozoan parasites, likely due to difficulties posed by the lack of genetic manipulation and relatively small scale of these cells. In *Entamoeba histolytica*, the intestinal protozoan that causes invasive amebiasis, the ER was first thought to be composed of vesicles of varying size but recently shown to be a continuous network (Teixeira and Huston, 2008). In *Toxoplasma gondii*, the infectious agent resulting in toxoplasmosis, expansion and partitioning of the ER has been followed during the cell cycle (Nishi et al., 2008). The ER in *Plasmodium* parasites, the causative agents of malaria, has been visualized during the erythrocytic cycle (van Dooren et al., 2005); it transforms from a perinuclear structure with no distinctive morphological characters into a reticular network throughout the cytoplasm. *Plasmodium* develops first in the *Anopheles* mosquito, and then invades the liver upon injection into mammalian hosts. Whether the ER adopts its characteristic shapes in these stages is yet to be determined, and the molecular determinants of the ER in *Plasmodium* parasites remain unclear.

To identify *Plasmodium falciparum* orthologs of ER-shaping proteins, we conducted Blast searches of the *P. falciparum* genomic database (PlasmoDB, www.plasmoDB.org, v. 6.3, released December 22, 2009) using *Saccharomyces cerevisiae* Yop1p (*Sc*Yop1p) and human DP1 protein sequences as queries. Both searches revealed 35%–42% identity (55%–60% homology) with PFC0730w (Gene ID PF3D7_0316700 in PlasmoDB v.26, released October 15, 2015). The PFC0730w sequence was further used to query *P. berghei* proteins using the tblastn tool. This search returned PBANKA_0414500 and PBANKA_1135000 as the closest *P. berghei* homologs (Fig. S1). PBANKA_0414500 was recently annotated as a putative HVA22/TB2/DP1 family protein and PBANKA_1135000 as a HVA22-like protein. Because HVA22 is the Yop1p homolog in plants and TB2 is a previously used alias for DP1, our homologous searches confirm the annotation of the genomic database. We renamed PBANKA_0414500 as *Pb*YOP1 and PBANKA_1135000 as *Pb*YOP1L (Fig. 1A and 1B). When searching for the reticulon homolog in the *P. berghei* genome, we found PBANKA_1139900 and renamed it *Pb*RTN1 (Fig. 1B). According to PlasmoDB database, the three potential *Plasmodium* tubule-forming proteins have distinct expression profiles during the asexual cycle: the expression of *Pb*YOP1 gradually increases and peaks in trophozoites and schizonts, the levels of *Pb*YOP1L peak in rings and trophozoites but decrease in schizonts, and levels of *Pb*RTN1 are relatively constant. These data suggest non-redundant functions of these proteins.

**Figure 1:**
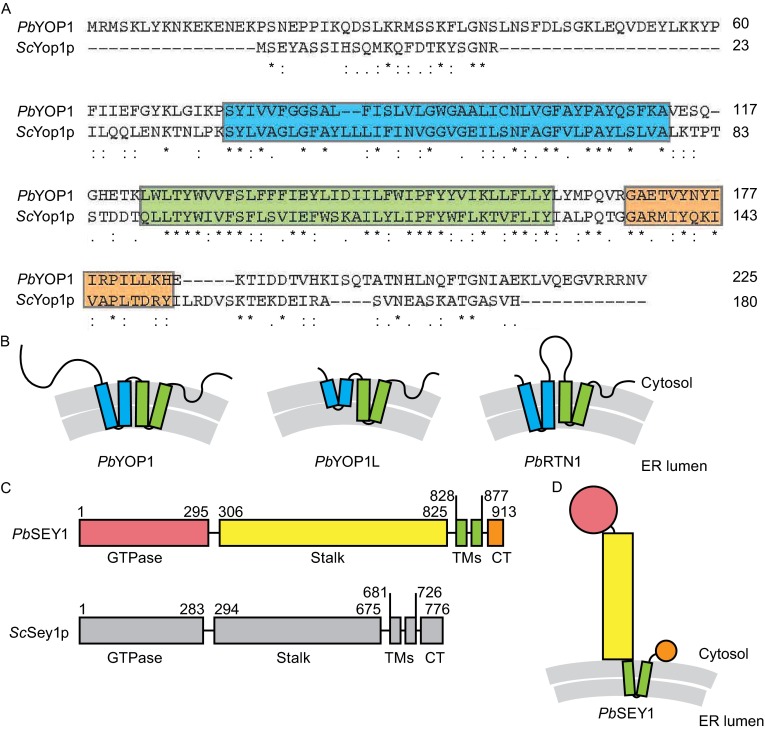
Identification of *Plasmodium* ER-shaping proteins. (A) Sequence alignment of *P. berghei* YOP1 (*Pb*YOP1) and *S. cerevisiae* Yop1p (*Sc*Yop1p). Residues are numbered; identical residues are indicated by asterisks and similar residues by dots. The first transmembrane segment is highlighted in cyan, the second in green, and the predicted C-terminal amphipathic helix in orange. (B) Topology diagrams of three ER tubule-forming proteins identified in the *P. berghei* genome. Lipid bilayers are shown in gray, the first transmembrane segment in cyan, and the second one in green. The length of each transmembrane segment and loop is shown in proportion to the predicted length. (C) Domain diagrams of *P. berghei* SEY1 (*Pb*SEY1) and *S. cerevisiae* Sey1p (*Sc*Sey1p). Domain boundaries are labeled. TM, transmembrane segment; CT, C-terminal tail. (D) Topology diagram of *Pb*SEY1. The domains are colored as in (C)

Thus, three potential ER tubule-forming proteins were identified in *P. berghei*. A common feature of ER tubule-forming proteins is a reticulon-homology domain (RHD) consisting of two tandem transmembrane hairpins (TMH) (Fig. 1B). The sequences of all three candidates for ER tubule formation in *P. berghei* exhibited characteristics of the RHD domain. Notably, the intervening loop between the two TMHs in *Pb*RTN1 is much longer than that of Yop1 family proteins, including *Pb*YOP1 and *Pb*YOP1L (Fig. 1B). In contrast, *Pb*YOP1 bears a much longer N-terminus than *Pb*YOP1L and *Pb*RTN1. The length of the TMHs is also variable, with the first TMH of *Pb*YOP1L and the second TMH of *Pb*RTN1 being shorter than other TMHs (Figs. 1B and S2), suggesting that it partially traverses the lipid bilayer. Importantly, the primary structure of *Pb*YOP1 is highly conserved among *Plasmodium* species (Fig. S2), implying a fundamental role of the protein.

We performed a similar search for the homolog of Sey1p in the *P. berghei* genome and identified PBANKA_1026600 as *Pb*SEY1 (Fig. 1C and 1D). Four conserved motifs were identified in the GTPase domains of the dynamin superfamily: the P-loop (G1, β1–α1), the switch 1 region (G2, α1–β2), the switch 2 region (G3, β3–α2), and the G4 motif (after β5). Most residues in these signature motifs are identical between *Sc*Sey1p and *Pb*SEY1 (Fig. S3). The predicted stalk domain of *Pb*SEY1, which is between the GTPase domain and the transmembrane domains, is ~150 amino acids longer than that of *Sc*Sey1p and ~400 amino acids longer than those found in ATLs. The length of the stalk domain of *Sc*Sey1p has been shown to be essential for fusion (Yan et al., 2015) and to possibly contribute to the unique GTP cycle of *Sc*Sey1p when compared to ATL. If this region forms a helical bundle as predicted, it would be taller than that of *Sc*Sey1p, implying likely different dynamics during membrane fusion reactions. Taken together, the ER-shaping proteins identified in *P. berghei* may function similarly to those of yeast and mammalian orthologs and may possess unique features.

Purified *Sc*Yop1p, when reconstituted into proteoliposomes, leads to the generation of membrane tubules *in vitro*, indicating that Yop1p is sufficient to induce high curvature in membranes (Hu et al., 2008). To test whether *Pb*YOP1 is capable of bending membranes as demonstrated for *Sc*Yop1p, we performed *in vitro* reconstitution assays. Recombinant *Pb*YOP1 (residues 39–186) lacks the predicted flexible region in the N- and C-termini for optimized expression and stability. *Pb*YOP1 was expressed and purified from *Escherichia coli* (Fig. 2A), mixed with preformed liposomes, and detergents were removed by the addition of bio-beads. When the reconstituted products were visualized by transmission electron microscopy (TEM), tubular structures similar to those of *Sc*Yop1p and *Sc*Rtn1p were observed (Fig. 2B). The diameters of these tubules are relatively uniform (15–20 nm), which is consistent with the *Sc*Yop1p tubules. In the absence of *Pb*YOP1, liposomes exhibited a characteristic round shape (Fig. 2B). These results suggest that *Pb*YOP1 alone is adequate to induce high curvature in membranes and cause tubule formation upon reconstitution with lipids.

**Figure 2:**
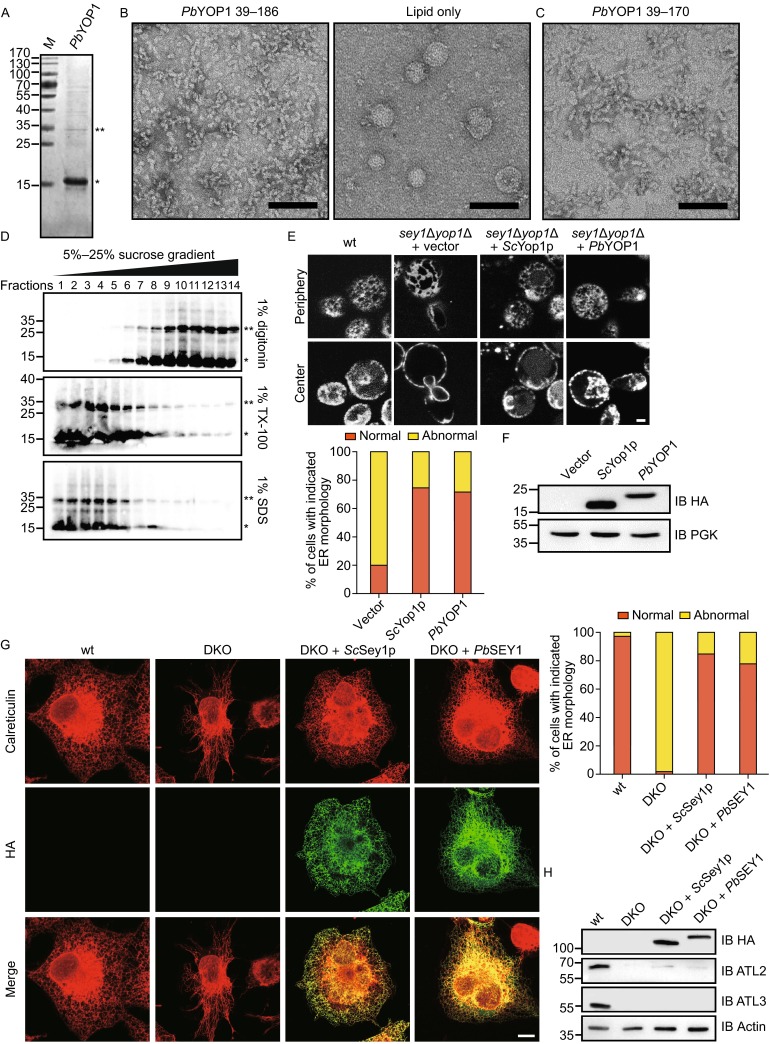
Functional tests of *Pb*YOP1 and *Pb*SEY1. (A) Purified *Pb*YOP1-HA (residues 39–186) was analyzed by SDS-PAGE and coomassie staining. SDS-resistant dimers are indicated by asterisks. Molecular marker is shown in kDa. (B) *Pb*YOP1-HA was mixed with *E. coli* polar lipids in Foscholine-12 and the detergent removed with Bio-beads over 4 h. The proteoliposomes were analyzed by negative stain EM. The right panel shows the sample with lipid only. Scale bar = 200 nm. (C) As in (B), but with *Pb*YOP1 lacking the C-terminal amphipathic helix (residues 39–170). (D) Sucrose gradient centrifugation of reconstituted *Pb*YOP1 treated with the indicated detergents. *Pb*YOP1-HA in 1% digitonin (top panel), 1% Triton X-100 (middle panel), or 1% SDS (bottom panel) was loaded onto a sucrose step gradient. After centrifugation for 2 h at 25°C, fractions were analyzed by SDS-PAGE and immunoblotting with HA antibodies. Detergent-resistant dimers are indicated by asterisks. (E) A RFP targeted to the ER lumen (ss-RFP-HDEL) was expressed in wild type (wt) or *sey1Δ* *yop1Δ* yeast cells. The localization of the protein was determined by fluorescence microscopy focusing at the center or periphery of the cells. Empty vector or indicated SEY1s were also expressed. The ER morphology was determined by counting at least 100 cells for each sample. The results are representative of at least three repetitions. Scale bar = 2 μm. (F) Indicated SEY1s expressed in *sey1Δ* *yop1Δ* cells were determined by anti-HA immunoblotting. PGK was used as a loading control. (G) ATLs-deleted COS-7 cells (DKO) were transfected with HA-tagged SEY1s. The ER morphology of indicated cells was visualized using calreticulin, an endogenous luminal ER protein, with indirect immunofluorescence and confocal microscope, and categorized as “normal” or “abnormal”. A total of 80–100 cells were counted for each sample. All graphs are representative of three repetitions. Scale bar = 10 μm. (H) ATL2, ATL3, and SEY1s levels were determined by immunoblotting. Actin was used as a loading control

The two TMHs of the RHD domain are thought to occupy more space in the outer leaflet than the inner leaflet of the lipid bilayer, deforming membranes as a wedge insertion. A conserved amphipathic helix C-terminal to the second TMH of *Sc*Yop1p was recently identified to provide additional wedging (Brady et al., 2015). Deletion of the helix in *Sc*Yop1p abolished its tubule-forming ability *in vitro*. Through secondary structure prediction and helical wheel analysis, we found two potential helices that exhibit an amphipathic nature, one on each side of the RHD domain of *Pb*YOP1. To test the role of these helices, we synthesized a peptide corresponding to the sequences of the C-terminal helix (Fig. S4A, residues 171–184) and determined its membrane association as expected for other membrane-deforming amphipathic helices. Circular dichroism spectrum analysis revealed that the peptide only adopted an α-helical configuration when liposomes were present (Fig. S4B), indicating stabilization of the peptide in a membrane environment. To our surprise, mutant *Pb*Yop1 (residues 39–170) missing the entire C-terminus, including the predicted amphipathic helix, could still form tubules *in vitro* (Fig. 2C). Therefore, we investigated the role of the N-terminal helix. We found that deletion of the N-terminal region of *Pb*YOP1 did not yield any usable recombinant protein (Fig. S4C). Instead, we obtained a major contaminant after attempting to purify truncated *Pb*YOP1 (residues 73–186). When the contaminant underwent the same reconstitution process, no tubular structures were formed (Fig. S4D), further confirming that the tubules we observed could only be specifically generated by functional *Pb*YOP1 proteins. As an alternative, we generated point mutations in the predicted hydrophobic face of the helix. However, mutant *Pb*YOP1 (residues 39–186, L45D/L49D/V52D) formed tubules like the wild-type protein when reconstituted with lipids (Fig. S4D). Taken together, these results suggest that the C-terminal region of the RHD domain is dispensable for generating membrane tubules and the N-terminus is important for the stability of the protein but may not function as an amphipathic helix to augment wedge insertion.

In addition to the “wedge” mechanism, *Sc*Yop1p and *Sc*Rnt1p can form homo- and hetero-oligomers through which these proteins may act as arc-like scaffolds to shape the membrane tubules (Shibata et al., 2009). We tested the oligomerization tendency of *Pb*YOP1 using a sucrose-density gradient. When reconstituted *Pb*YOP1 was solubilized by digitonin, a relatively mild detergent, it migrated in the gradient at a position that corresponds to a much higher molecular weight than that of a monomer (Fig. 2D). The oligomerization was largely disrupted when Triton X-100 or SDS was used instead (Fig. 2D). Notably, SDS-resistant dimers were often observed when PbYOP1 was separated by SDS-PAGE and analyzed by immunoblotting (Fig. 2D). These results confirm that *Pb*YOP1 forms oligomers.

To further confirm the ER-shaping function of *Pb*YOP1, we performed ER morphology rescue assays in yeast cells. When Sey1p, the ER fusogen in yeast, and Yop1p or Rtn1p are deleted, the peripheral ER (also termed cortical ER in yeast because it localizes underneath the plasma membrane) displays abnormal morphology. Large areas of the cortex are void of ER structures and most of the tubular ER network becomes sheet-like; the re-introduction of either Yop1p or Rtn1p in these cells restores the ER morphology (Hu et al., 2009). To ensure the expression of *Pb*YOP1 in yeast, we utilized a 2μ vector with high copy numbers (pESC-URA) and drove protein expression using an inducible GAL promoter. The coding region of *Pb*YOP1 was optimized for yeast codon usage to achieve detectable expression. As expected, most *sey1Δyop1Δ* cells expressing *Pb*YOP1 exhibited normal ER morphology (Fig. 2E). The *Pb*YOP1 levels were similar to *Sc*Yop1p expressed under control of the endogenous promoter (Fig. 2F). These results suggest that *Pb*YOP1 can replace *Sc*Yop1p in maintaining proper ER morphology.

Next, we performed ER morphology rescue assays in mammalian cells and tested the function of *Pb*SEY1. ER tubules become long and unbranched when ATL2 and ATL3 are deleted using CAS9/CRISPR system in COS-7 cells (ATL1 is not detectable in COS-7 cells) (Hu et al., 2015; Wu et al., 2015), indicative of the lack of fusion between ER tubules (Fig. 2G and 2H). As previously reported (Yan et al., 2015), when *Sc*Sey1p were expressed in these cells, the defects in ER morphology were largely restored (Fig. 2H). *Pb*SEY1 do not express in yeast or mammalian cells, likely due to different codon usage. When codon-optimized *Pb*SEY1 was expressed in ATL-deleted COS-7 cells, a majority of the mutant cells exhibited normal tubular ER network (Fig. 2H). These results confirm that *Pb*SEY1 is functionally analogous to its yeast or mammalian orthologs.

Our findings reveal four ER-shaping proteins in the *P. berghei* genome. *Pb*YOP1, *Pb*YOP1L, and *Pb*SEY1 have not been characterized previously. We showed that, similar to *Sc*Yop1p, *Pb*YOP1 generated membrane tubules when purified and reconstituted *in vitro*, even though a conserved amphipathic helix next to the transmembrane domain is less important than in *Sc*Yop1p. We also confirmed the role of *Pb*YOP1 and *Pb*SEY1 in maintaining the tubular ER network in cells. Although the ER shaping activities of these proteins need to be further tested in *Plasmodium*, our results imply that the ER of *Plasmodium* parasites likely forms a tubular network via common mechanisms as described for yeast and mammalian cells. In addition, the ER-shaping proteins of *Plasmodium* parasites possess unique features that may meet specialized demands on the parasitic ER. The homolog of *Pb*RTN1 in *Plasmodium yoelii nigeriensis* has been termed Pyn_chl091 due to its transcriptional up-regulation in mosquito vector when treated with chloroquine, an anti-malarial drug (Silveira et al., 2007). These ER-shaping proteins, including *Pb*RNT1, likely adopt specialized roles during stressed conditions.

Very little is known about the morphology and function of the *Plasmodium* ER, especially during the pathogenic path of the parasites. Identification and preliminary characterization of *Plasmodium* ER-shaping proteins lays the foundation for further investigation of the *Plasmodium* ER. A lack of individual ER-shaping proteins in unicellular organisms, such as yeast, causes no drastic defects. However, in *Candida albicans*, an infectious fungus, deletion of Sey1p decreases its virulence (Yamada-Okabe and Yamada-Okabe, 2002), leading to the possibility that ER morphology determinants play a role in the infection of protozoan parasites, including malaria.

## Electronic supplementary material

Below is the link to the electronic supplementary material.

Supplementary material 1 (PDF 8846 kb)
