# Supplementary material for: Identification of endoplasmic reticulum-shaping proteins in Plasmodium parasites
Source: Protein Cell. 2016 Aug 2;7(8):615–20. doi: 10.1007/s13238-016-0290-5 (PMC4980331; doi:10.1007/s13238-016-0290-5)
Supplement: Supplementary file 1 — Supplementary material 1 (PDF 8846 kb) [file 13238_2016_290_MOESM1_ESM.pdf]

**Electronic supplementary material**

## Materials and methods

### Constructs

*Pb*YOP1 was PCR-amplified from the genome of *P. berghei* ANKA. The intron was removed using overlapping PCR, and the resulting coding region with a C-terminal HA-tag was ligated into pET28a plasmid via the NheI/Xhol1 sites. Mutation of *Pb*YOP1 was performed using the QuikChange Lightning Site-Directed Mutagenesis Kit (Stratagene). For yeast expression, *Pb*YOP1 was amplified and inserted into pESC-URA. All constructs were verified by sequencing.

### Protein purification

Plasmids expressing wild-type or mutant His-*Pb*YOP1-HA were transformed into DE3 strains and cultured in 1L Luria Broth medium. IPTG (300 μM) was added when the cultures reached an OD600 of 0.8 and grown overnight at 16˚C. Cells were harvested, resuspended in TSG (50 mM Tris pH 8.0, 150 mM NaCl, 20 mM imidazole, 10% glycerol), and lysed by sonication. The lysates were then mixed with 1% Foscholine-12 (Anatrace) for 1 h and cleared by centrifugation for 1 h at 4˚C in a rotor (45 Ti; Beckman Coulter) at 30,000 rpm. The supernatant was then incubated with 1 mL Ni-NTA sepharose (GE healthcare), washed with TSG containing 0.1% Foscholine-12, and eluted with TSG containing 300 mM imidazole and 0.1%

Foscholine-12. The elution was collected and concentrated to 500 µL and 30U thrombin added to remove the His-tag overnight. The protein was then further purified by gel filtration (Superdex 200, GE healthcare) in TSG containing 0.1% Foscholine-12. Selected fractions were concentrated to 2 mg/mL for reconstitution.

**Reconstitution**

For SUV production, POPC and DOPS (Avanti Polar Lipid) were mixed at a molar ratio of 85:15 (10 mM of total lipid). The mixture was then dried under a stream of $N_2$ and rehydrated in TSG1 buffer (50 mM Tris pH 8.0, 150 mM NaCl, 10% glycerol). After 10 freeze-thaw cycles, the vesicles were then extruded 11 times through the extruder (Avanti Polar Lipid) with 100 nm or 400 nm filter membranes. Purified *Pb*YOP1 (final concentration 0.4 mg/mL) in Foscholine-12 was then mixed with the pre-formed SUV (final concentration 0.4 mM) for 30 min at room temperature. The detergent was removed by addition of SM-2 Bio-beads four times at room temperature. Finally, the proteoliposomes were cleared by centrifugation at 14,000 rpm for 10 min.

**Electron microscopy**

Negative staining was performed with 2% uranyl acetate. First, 5 µL of the 5X diluted proteoliposome sample was placed onto a carbon-coated copper grid for 1 min, the excessive sample dried by filter paper, and the grid washed with deionized water. A total of 5 µL of filtered 2% uranyl acetate was placed on the grid and

excessive stain dried by filter paper. Images were collected at room temperature using a Hitachi TEM system operated at an acceleration voltage of 100 kV. Images were recorded at a magnification of 30,000 and a defocus value of 1.5 μm. All images were recorded on a 2k x 2k CCD camera.

**Sucrose gradient centrifugation**

A 5-layer sucrose gradient (5%, 10%, 15%, 20%, 25%, 50 μL each) in TS buffer (50 mM Tris pH 8.0 and 150 mM NaCl) was prepared in centrifugation tubes. Proteoliposomes treated with 20 μL 1% digitonin, Triton X-100, or SDS were loaded onto the top layer of the gradient. The samples were then centrifuged at 174,000 rpm in a TLS-55 rotor (Beckman Coulter) for 2 h at 4°C. Each fraction sample was analyzed by Western blot with HA-tag antibody.

**Circular dichroism**

Synthesized peptide (50 μM) in 10 mM potassium phosphate (pH 7.5) was mixed with TSG buffer and 100 nm SUV (1 mM lipids) or 400 nm SUV (1 mM lipids) separately. Circular dichroism was performed on a Biologic MOS-450 instrument at room temperature. Spectra were collected from 190-260 nm at a bandwidth of 1 nm.

**Mammalian cell culture and transfection**

COS-7 cells (American Type Culture Collection) were maintained in complete Dulbecco's minimum essential medium supplemented with 10% fetal bovine serum

in 5% $CO_2$ at 37°C. Transfections were performed using Turbo (Thermo) according to the manufacturer's instructions. To generate ATL-deleted cells by CRISPR/Cas9 genome editing, guide RNA (gRNA) sequences were designed using the CRISPR design tool as following, with protospacer adjacent motifs (PAMs) underlined:

*ATL2*: 5′-GACGAGATCTTAACATAGTAGTGG-3′.

*ATL3*: 5′-GTTTTCACTGTGGAGAAGCCAGG-3′.

gRNA containing oligonucleotides were introduced into the pX330 vector. CRISPR/Cas9 plasmids were transiently transfected into COS-7 cells along with pLKO.1-puro at 1:1:1 ratio using TurboFect transfection reagent (Thermo). 24 hours later, transfected cells were selected with 1 μg/ml puromycin for 1 week. The cells were then sorted for single cell into a 96 well plate by a BD FACS Aria-II sorter. Different single clones were verified by immunoblotting and sequencing.

**Immunofluorescence and confocal microscopy**

Indirect immunofluorescence of paraformaldehyde-fixed cells was described previously (Hu et al., 2009). Transfected cells were grown on coverslips and immunostained with anti-calreticulin (abcam; 1:500) and anti-HA antibodies (Sigma; 1:500) as primary antibodies and various Alexa Fluor-conjugated secondary antibodies (AlexaFluor 488 anti-mouse or AlexaFluor 568 anti-rabbit, 1:1000, Invitrogen). Yeast cells were imaged live as described previously (Hu et al., 2009). All images were captured on an OLYMPUS FV1200 confocal microscope with a

60×/1.40 NA Plan Apochromat oil immersion objective lens using the Olympus Fluoview Version 2.0b Viewer. Brightness and contrast were adjusted across the entire image using Adobe Photoshop.

```
PbYOP1L         ------------------------------------------------------------
PbYOP1          MRMSKLYKNKEKENEKPSNEPPIKQDSLKRMSSKFLGNSLNSFDLSGKLEQVDEYLKKYP
HVA22           ------------------------------------------------------------

PbYOP1L         ----------------MGLHIFPTKIVNLVNIIVSIFCPAAETYNLLF-HKKDKANEDYV
PbYOP1          FIIEFGYKLGIKPSYIVVFGGSALFISLVLGWGAALICNLVGFAYPAYQSFKAVESQ-GH
HVA22           ----------------------MGKSWALLTHLHSVAGPSITLLYPLYASVCAMESPSKV
                                                  ::           :       .

PbYOP1L         HHIHFITYWIIYSLYYCLESLLLIHIMNYIPFYFELKLLLFFWLYNDTFQGAGYIYFKFI
PbYOP1          ETKLWLTYWVVFSLFFFIEYLI-DIILFWIPFYYVIKLLFLLYLYMPQVRGAETVYNYII
HVA22           DDEQWLAYWILYSFITLLEMVA-EPVLYWIPVWYPVKLLFVAWLALPQFKGASFIYDKVV
                .   :::**:::*:   :* :    :: :**.:: :***:. :*    .:**  :*  .:

PbYOP1L         EKYYSTIDKKICDIV----YTN---VPKNIINLFPFEKQQPITIKKSSSKLRSMVSK
PbYOP1          RPILLKHEKTI-----DDTVHKISQTATNHLNQFTGNIAE--KLVQEGVRRRNV---
HVA22           REQLRKYRGRNRNGDADHKVHILKAEADH------GRVH------------------

                .    .                      .       .
```

Figure S1. Sequence alignment of *Pb*YOP1, *Pb*YOP1L and HVA22. Labeling is as in Fig. 1A.

```
PBANKA_0414500      MRMSKLYKNKE--KENEKPSNEPPIKQDSLKRMSSKFLGNSLNSFDLSGKLEQVDEYLKK
PCHAS_0415400       MRMGKLYKNKDK--ENDKSGSDSPNKQDSLKRMSSKLLGNSLNSFDIGGKLDQLDEYLKK
PY17X_0417300       MRMSKLYKNKEKEKENEKPSNEPITKQNSLKRMSSKILGDSLNSFDLSGKLEQVDEYLKN
PYYM_0417300        MRMSKLYKNKEKEKENEKPSNEPITKQNSLKRMSSKILGDSLNSFDLSGKLEQVDEYLKN
PFIT_0316700        MKMTKLYKHKEKE-------DRPNTSLNSLKRISSSVFGEKLNNLDVSRVFNNIDDYVKK
PF3D7_0316700       MKMTKLYKHKEKE-------DRPNTSLNSLKRISSNVFGEKLNNLDVSRVFNNIDDYVKK
PRCDC_0316000       MKMTKLYKHKEKE-------DRPNTSLNSLKRISSNVFGEKLNNLDVSRVFNNIDDYVKK
PKNH_0825100        MKGKLYSKHKDKDNEKYG-AGGSSQNINALKRLSSKVLGDSINYFDLNKTLDKIDEHVKQ
PCYB_083300         MKGKLYSKSKDKEHEKYG-GGGSSQNMNALKRLSSKVLGDSINNFDLSKTLDKIDEHVKQ
PVX_095400          MKGKLYPKNKDKEHERYG-GGGGSQNMSALKRLSSKVLGDSVSNFDLSKTLDKIDEHVKQ
                    *:     * *:              . .:***:**..:*:.:. :*:   ::::*:::*:

PBANKA_0414500      YPFIIEFGYKLGIKPSYIVVFGGSALFISLVLGWGAALICNLVGFAYPAYQSFKAVESQG
PCHAS_0415400       YPFIIEFGHKLGIKPSCLVVFGGSIVFISLVFGWGAALICNLVGFAYPAYQSFKAVESQG
PY17X_0417300       YPFIIEFGYKIGIKPSYIVVFGGSALFISLVLGWGAALICNLVGFAYPAYQSFKAVESQG
PYYM_0417300        YPFIIEFGYKIGIKPSYIVVFGGSALFISLVLGWGAALICNLVGFAYPAYQSFKAVESQG
PFIT_0316700        YPFLNNIGKKFGVKPSYIIVPFSVFLFLSLVFGWGAAIICNVVGFAYPAYQSFKAVESQS
PF3D7_0316700       YPFLNNIGKKFGVKPSYIIVPFSVFLFLSLVFGWGAAIICNVVGFAYPAYQSFKAVESQS
PRCDC_0316000       YPFLNNIGKKFGVKPSYIIVPFSVFLFLSLVFGWGAAIICNVVGFAYPAYQSFKAVESQS
PKNH_0825100        YPFLDDMGKKYGIKPSYVVVGFGGFLLLSLIFGWGAALICNVVGFAYPAYQSFKAVESQC
PCYB_083300         YPFLDDLGKKYGIKPSYVIVGMSGFLFLSLIFGWGAALICNVVGFAYPAYQSFKAVESQS
PVX_095400          YPFLDDLGKKYGIKPSYVIVGVSGFLFLSLIFGWGAALICNVVGFAYPAYQSFKAVESQR
                    ***: ::* * *:*** ::*  .  :::**::*****:***:*****************

PBANKA_0414500      HAETKLWLTYWVVFSLFFFIEYLIDIILFWIPFYYVIKLLFLLYLYMPQVRGAETVYNYI
PCHAS_0415400       HAETKLWLTYWVVFSLFFFIEYLIDIILFWIPFYYVIKLLFLLYLYMPQVRGAETVYNYV
PY17X_0417300       HAETKLWLTYWVVFSLFFFIEYLIDIILFWVPFYYVIKLLFLLYLYMPQVRGAETVYNYV
PYYM_0417300        HAETKLWLTYWVVFSLFFFIEYLIDIILFWVPFYYVIKLLFLLYLYMPQVRGAETVYNYV
PFIT_0316700        RDETKLWLTYWVVFSLFFFIEYLIDIILFWVPFYYLIKLLFLLYLYMPQVRGAVMVYNYI
PF3D7_0316700       RDETKLWLTYWVVFSLFFFIEYLIDIILFWVPFYYLIKLLFLLYLYMPQVRGAVMVYNYI
PRCDC_0316000       RDETKLWLTYWVVFSLFFFIEYLIDIILFWVPFYYLIKLLFLLYLYMPQVRGAVMVYNYI
PKNH_0825100        KDETKLWLTYWVVYSLFFFFEYLIDIILFWVPFYYLLKLLFLLYLYMPQVRGAETVYNYI
PCYB_083300         KDETKLWLTYWVVYSLFFFFEYLIDIILFWVPFYYLLKLLFLLYLYMPQVRGAETVYNYI
PVX_095400          KDETKLWLTYWVVYSLFFFFEYLIDIILFWVPFYYLLKLLFLLYLYMPQVRGAETVYNLV
                    : ***********:*****:**********:****::****************  *** :

PBANKA_0414500      IRPILLKHEKTIDDTVHKISQTATNHLNQFTGNIAEKLVQEGVRRRNV
PCHAS_0415400       IRPVLLKHEKTIDDTVHKISQTATSHLNQITGNIADKIVQEGVRRRNV
PY17X_0417300       IRPILLKHEKAIDDTVHKISQTATNHLNQFTGNIAEKLVQEGVRRRNV
PYYM_0417300        IRPILLKHEKAIDDTVHKISQTATNHLNQFTGNIAEKLVQEGVRRRNV
PFIT_0316700        IRPILLKHEKMIDDTVQKISQTATSHLTQITGNLTEKLVQEGIRRRHI
PF3D7_0316700       IRPILLKHEKMIDDTVQKISQTATSHLTQITGNLTEKLVQEGIRRRHI
PRCDC_0316000       IRPILLKHEKMIDDTVQKISQTATSHLTQITGNLTEKLVQEGIRRRHI
PKNH_0825100        IRPILLKHEKTIDDTVQKISQTATSHLTQITGNLTEKLVQEGVRRRNI
PCYB_083300         IRPILLKHEKAIDDTVQKISQTATSHLTQITGNLTEKLVQDGVRRRNV
PVX_095400          VRPILLKHEKTIDDTVQKISQTATSHLTQITGNLTEKLVQDGVRRRNV
                    :**:****** *****:*******.**.*:***:::*:**:*:***.:
```

Figure S2. Sequence alignment of *Plasmodium* homologs of *Pb*YOP1. Labeling is as in Fig. 1A.

```
PbSey1      MTDVNKTQIIDYDGHIIDN-LKEWMNNNKLSKLGFNYNVIAILGSQSSGKSTLLNNLFKT 59
ScSey1      MADRPAIQLIDEEKEFHQSALQYFQQCIGNRDVGLDYHVISVFGSQSSGKSTLLNVLFNT 60

PbSey1      SFDVMNTKLGHSQTTQGLWLSYDKFEDELTDASNEETDVEPQNKSNNKHVINPTLILDVE 119
ScSey1      NFDTMDAQVKRQQTTKGIWLAHTKQVNTTIEIDND---------------RPDIFVLDVE 105

PbSey1      GNDSKERGENRLTFEHRSALFSLALADCVIVNLWYHSLGNFTASNYGLLKTVMEVHLELF 179
ScSey1      GSDGSERGED-QDFERKAALFAIAVSEVLIVNMWEQQIGLYQGNNMALLKTVFEVNLSLF 164

PbSey1      HQNVNCPKTILLFTVRDWFEEFAPLDIIREKIVDEYVNKIWCELKKSENSKNANIDDYFI 239
ScSey1      GKNDNDHKVLLLFVIRDHVGV-TPLSSLSDS-VTRELEKIWTELSKPAGCEGSSLYDYFD 222

PbSey1      IEVVGLSHGIIKKDEFLKDIKRLRHKWIYELR-----PINYSRNIPADGFAQYCNNIWNT 294
ScSey1      LKFVGLAHKLLQEDKFTQDVKKLGDSFVMKGTENYYFKPQYHHRLPLDGWTMYAENCWDQ 282

PbSey1      IIKQSQLDIPSQQEMLATFRCQEIKNNVLNHISNTIKEKMVDSKNKYIENFKTWAEKDII 354
ScSey1      IERNKDLDLPTQQILVARFKTEEISNEALEEFISKYDESIAPLKGNLGS-L-TSQLVKLK 340

PbSey1      EKSLNEYLTDAARYQKIICLKTLEELLENLFIQLQIIVDNNLNFTQRILSSKFSKELNSM 414
ScSey1      EECLTKYDEQASRYARNVYMEKREALNTKLN-------------------SHISGTINEF 381

PbSey1      YSICTTDKSYFLFINDQNVDVTEQDENLSNVENIGENSKKGNQIKCINLWSNFLYNADML 474
ScSey1      L----------------------------------------ESLMEKLWDDLKLEVSSR 400

PbSey1      EYTT------------------IANFFDQYKKCSVEIIEPSISNNEDKDSQEKRNHDFNYK 517
ScSey1      DKATTSFVESVAAGKSKIEKEFNESMETFKKLGLLISNEEITCK---------------- 444

PbSey1      NSLTILATSIYKDTNRIRSVQCNILIERIRSTIKEELKNVDNMLITVKCSKDCWDYILKI 577
ScSey1      -----FSDDIEERIKQLRDAELKAKIGRIKKNLVPELKDHVI-HLLSHPSKKVWDDIMND 498

PbSey1      VNKLEDYIYTNLSKCFINLKTGINTTYLNNGDNIYARLNTNCDYGLGYFQNEQITDFSDD 637
ScSey1      F---ESTIKDNIS--------------------AYQVEKDKYDFKIGLSES--------- 526

PbSey1      AGKNDDEMDTEIDQNKNDMESLFNSKKFEIITKQNKKEKYVSTINNDLNKEMNNKKLISE 697
ScSey1      -----------------------------------------------------------E 527

PbSey1      LKKFYTEIIIDALK---IK--LDEISNNIANIIINRFESVFNYDEIDQPRQWRNISAIEL 752
ScSey1      NAKIYKNIRILAWRTLDTTVHDYLKIDTIVSILRDRFEDVFRYDAEGSPRLWKTEEE--I 585

PbSey1      KNIFRVSKDYAFLIIEILQKNIKIDKIDNYLSTNFINTDIIEKGKI-------KAKKRIQ 805
ScSey1      DGAFRVAKEHALEVFEVLSLAVTSDNVEIIPDVPMAEEESGEDNEIYRDNEGVFHSRRFA 645

PbSey1      EIC------------RDAQYIQ----ETGGHMSLKNVPFAFWVILLILGWNEILMFTRLF 849
ScSey1      HILTELQKENVLDQFRRQINITVLDSKRSIITTRTHIPPWIYVLLAVLGWNEFVAVIRNP 705

PbSey1      FRLNIILPMLIGFIIIVI--SCLYTGNAQILS-YINKIIFIVIKNLYNFYKHLQTIGHQT 906
ScSey1      LFVTL--TLILGATFFVIHKFGLWGPVVNVVQSAVGETRTAIKDKLRQFV----VEDHEV 759

PbSey1      TKP---------EKVE-
ScSey1      KESFEMKDFSKNEQKEK
```

Figure S3. Sequence alignment of *Pb*SEY1 and *Sc*Sey1p. Predicted secondary structures are labeled and conserved elements are highlighted.

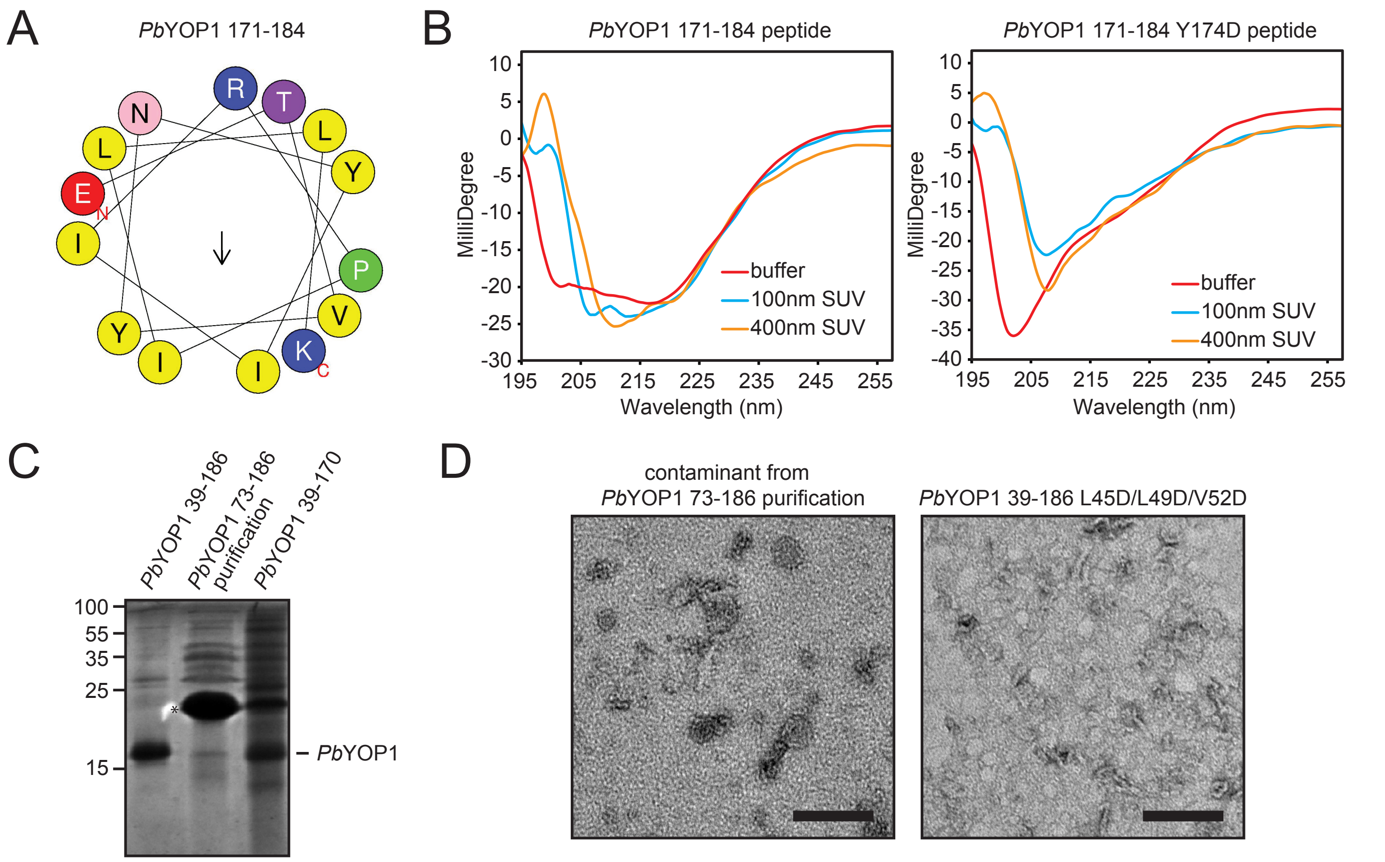


Figure S4. Amphipathic helices flanking the RHD domain of *Pb*YOP1. (A) Helical wheel analysis of the C-terminal helix. Hydrophobic residues are labeled in yellow. (B) CD measurements of wild type or Y174D mutant peptide in the absence or presence of liposomes as indicated. (C) Purification of *Pb*YOP1 mutants lacking the amphipahtic helices. Asterisk indicates a contaminant. Molecular marker is shown in kDa. (D) Reconsitututed proteoliposomes were analyzed by negative stain EM. Scale bar = 200 nm.
